# Single-Domain Antibodies—Novel Tools to Study and Treat Allergies

**DOI:** 10.3390/ijms25147602

**Published:** 2024-07-11

**Authors:** Ines Zettl, Clarissa Bauernfeind, Jessica Kollárová, Sabine Flicker

**Affiliations:** 1 Institute of Pathophysiology and Allergy Research, Center for Pathophysiology, Infectiology and Immunology, Medical University of Vienna, 1090 Vienna, Austria; 2 Center for Cancer Research, Medical University of Vienna, 1090 Vienna, Austria

**Keywords:** allergy, nanobody, VHH, allergy treatment, biologics, anti-IgE, type 2 cytokines, allergen surveillance

## Abstract

IgE-mediated allergies represent a major health problem in the modern world. Apart from allergen-specific immunotherapy (AIT), the only disease-modifying treatment, researchers focus on biologics that target different key molecules such as allergens, IgE, or type 2 cytokines to ameliorate allergic symptoms. Single-domain antibodies, or nanobodies, are the newcomers in biotherapeutics, and their huge potential is being investigated in various research fields since their discovery 30 years ago. While they are dominantly applied for theranostics of cancer and treatment of infectious diseases, nanobodies have become increasingly substantial in allergology over the last decade. In this review, we discuss the prerequisites that we consider to be important for generating useful nanobody-based drug candidates for treating allergies. We further summarize the available research data on nanobodies used as allergen monitoring and detection probes and for therapeutic approaches. We reflect on the limitations that have to be addressed during the development process, such as in vivo half-life and immunogenicity. Finally, we speculate about novel application formats for allergy treatment that might be available in the future.

## 1. Introduction

Around 30 percent of the population worldwide suffers from IgE-mediated allergies [1]. The prevalence is increasing, as observed in children’s sensitization profiles from birth cohort studies around the world [2,3,4,5,6]. Along with the health aspect, allergies also represent an economic burden to society since inadequate treatment and underestimation of allergies cause lack of productivity in school as well as in jobs [7]. Mechanistically, both humoral and cellular components are involved in allergic diseases. Key players are allergen-specific IgE antibodies, which are bound to the high-affinity IgE receptor (FcεRI) on mast cells and basophils in sensitized individuals. Upon IgE-crosslinking by the respective allergens, these effector cells become activated and release inflammatory mediators (e.g., histamines) [8]. IgE also binds to its low-affinity receptor FcεRII (CD23) on various immune cells such as B cells and macrophages, facilitating allergen presentation to T cells and driving the progression of the disease [9]. Multiple forms of intervention are available for affected individuals currently, and new concepts are continuously being explored. First-line medication is represented by symptomatic treatment (including antihistamines) that is given independently of the sensitizing allergen and is cheap and easily accessible. However, most available drugs are still hampered by side effects such as drowsiness while only offering short-term improvement [10]. An increasingly successful alternative to manage allergies is allergen-specific immunotherapy (AIT), the only disease-modifying therapy to date, based on multiple injections of the respective symptom-causing allergen [11,12,13]. Besides the induction of regulatory immune cells (DC_regs_, T_regs_, B_regs_) and the reduction in T helper (T_H_) cell activity, an important mechanism behind the efficacy of AIT is the induction of allergen-specific, blocking IgG antibodies. These IgG antibodies shield IgE epitopes on the corresponding allergens, thereby inhibiting IgE-crosslinking on effector cells [11,12,13]. A large body of the literature supports the effectiveness of AIT for different allergens, including hymenoptera venom, grass pollen, birch pollen, house dust mites, and foods [14,15,16], yet there are also some drawbacks. First, it is a very time-consuming therapy, only showing success after three to five years [17,18,19]. Second, since patients are given the respective symptom-inducing allergen, they have to be monitored during and after administration, making it a laborious treatment. Third, there is the chance, although low, of experiencing dangerous side effects such as anaphylactic shock [20,21]. A further limitation of AIT is the potentially restricted efficacy regarding cross-reactive allergens. Multiple studies dealing with birch pollen allergy showed that this form of therapy could not reliably induce cross-reactive IgG antibodies in all patients [22,23,24,25]. However, a large portion of patients do not only react with the major birch pollen allergen Bet v 1 but also with homologous allergens from related trees and foods [26] and therefore need to be protected against these allergen sources as well.

To overcome the adverse effects of the aforementioned treatment approaches and provide immediate protection, passive immunization with allergen-specific monoclonal IgG antibodies has been the focus of allergologists in recent years. Recently, studies have shown the success of passive immunization for birch pollen and cat allergy, as patients did not show symptoms upon allergen challenge for up to three months after receiving a single dose of allergen-specific IgG antibodies [27,28,29,30]. The success of monoclonal antibodies is furthermore reflected in the number of therapeutics that are already approved or in clinical development acting on other steps of the allergic cascade such as IgE receptor binding or cytokine signaling. The use of monoclonal antibodies for allergy treatment has been covered recently in detail elsewhere [31].

However, the laborious and expensive generation of human(ized) monoclonal antibodies [32] has shifted the research emphasis to smaller antibody fragments, including single-domain antibodies, also known as nanobodies (nanobody^®^ is a trademark of Ablynx N.V.). Nanobodies derive from an uncommon type of antibody named “heavy-chain-only antibody” (HCAbs) which is present in the blood of camelids and certain cartilaginous fish [33,34,35,36,37]. They convince with their simple, single-domain architecture and, hence, easy production, prolonged stability, and high specificity and affinity for their target. Moreover, their simple structure allows them to be modified and used as building blocks for larger, multivalent, or multi-specific constructs, giving them tailored features to meet their specific requirements [38]. Manufacturing nanobodies is now a well-established protocol and can be streamlined easily. Researchers can choose from multiple platforms to generate and isolate their perfect candidate. Immune libraries require the immunization of camelids (camels, llamas, and alpacas) with the antigen of choice, extraction of PBMCs after several weeks, and cloning of the full cDNA repertoire using PCR amplification [39]. This sort of library usually yields 10^7^–10^9^ clones and represents affinity matured nanobodies. Recently, a research group managed to generate transgenic mice (LaMice) that produce llama HCAbs, whereas their endogenic VH genes are knocked out [40]. This attempt rekindled the interest in this sophisticated technology based on earlier efforts to create hybrid llama/human antibodies [41]. Since mice are easier to house and breed than camelids, this advanced model may facilitate and accelerate nanobody discovery in the future. Alternatively, synthetic libraries (comprising up to 10^12^ clones) are available to be screened directly for the desired specificity [42,43]. While the huge advantage in synthetic libraries lies in circumventing animal use and selecting nanobodies against toxic or endogenous targets, animal immunization leads to more specific and highly affine nanobodies. From these libraries, strong binders are usually isolated by phage or yeast display [38] (Table 1).

The combination of facile production and biophysical properties successfully established nanobodies in a variety of medical fields such as cancer, autoimmune diseases, and infectious diseases [61,62]. In 2018 and 2019, the first therapeutic nanobody Caplacizumab (trade name Cablivi), a bivalent construct targeting the van Willebrand factor (vWF) to treat thrombotic thrombocytopenic purpura, was approved by the EMA and FDA, respectively [63,64]. Very recently, lessons learned from five years of controlled trials and clinical experience from daily practice have been published, leading the way on the nanobody application road map [65].

In allergology, however, nanobody applications are scarce, despite their promising features. Nevertheless, we have observed an emerging wave of allergen-specific nanobodies in recent years. Within this review, we aim to summarize the data available on the application of nanobodies in allergy research and treatment, highlight the relevant characteristics required to qualify them for clinics, and provide an outlook on what we may expect in this field in the coming years.

## 2. In Vitro Characterization of Allergen-Specific Nanobodies

The path towards finding suitable allergen-specific nanobodies to study and treat allergies leads through a thorough in vitro characterization. Nanobodies have to be assessed for their specificity to their target allergen, their cross-reactivity to related allergens, and their kinetic properties such as affinity. These attributes are prerequisites to employ them for allergen detection and prediction, diagnosis, or for topical treatment of allergic reactions. Of note, nanobodies additionally need to be evaluated for their potential to block IgE–allergen binding when selected for passive systemic treatment. All these characteristics are crucial to identify effective candidates for further half-life and safety in vivo studies.

### 2.1. Importance of Cross-Reactivity to Related Allergens

Cross-reactivity is generally described as an immune-mediated phenomenon of antibodies to spot similar protein patterns in different antigens due to structural similarity between homologous proteins. In the field of allergy research, this property is of importance, as IgE antibodies of allergic patients often broadly cross-react with structurally similar allergens from related sources [26,66,67]. One of the best studied and documented allergens in terms of cross-reactivity is the major birch pollen allergen, Bet v 1. It is well known that extracts of alder, hazel, hornbeam, and oak contain Bet v 1 cross-reactive allergens that provoke the prolongation of allergy symptoms beyond the birch pollen season [26,68,69]. Additionally, a lot of patients with diagnosed birch pollen allergy also suffer from allergic symptoms after the consumption of certain foods, which is caused by cross-reactions between pollen and foods. This fact further potentiates allergic reactions and is known as pollen food allergy syndrome [70,71]. The described example illustrates the relevance of cross-reactivity between allergens and the importance of identifying allergen-specific nanobodies that are able to bind those cross-reactive allergens in order to develop an effective and protective treatment for allergic patients [72]. Currently, no standardized methods for cross-reactivity testing of allergens are available [73]. The most common methods for the identification of cross-reacting allergens are ELISA-based assay, ImmunoCAP, or ISAC. All three techniques have been frequently applied to determine specific IgE reactivity to immobilized cross-reactive allergens and, furthermore, to quantify specific IgE concentrations in serum and body fluids to define clinical phenotypes of allergies [74,75,76].

However, similar three-dimensional folding but also linear sequence homology are sometimes insufficient to predict true cross-reactivity. Therefore, ELISA and ISAC have also been employed to detect specific IgG binding. Due to the low availability of immobilized allergens these techniques are best suited to assess the potential of AIT-induced antibodies, monoclonal IgG antibodies, or IgG-derived antibody fragments to compete with IgE antibodies for allergen binding [27,49,52,77]. This insight was crucial because it turned out that direct inhibitory responses predict antibodies’ cross-protection, a fact that will be covered in more detail for nanobodies in Section 2.3.

The capacity to cross-react with homologous allergens was demonstrated for allergen-specific nanobodies for the first time recently. Nanobodies specific for Bet v 1 were shown to recognize homologs from related tree pollen [44]. When engineered as nanobody trimers, extended cross-reaction to Bet v 1 relatives of pollen-related food was observed [45]. Similar results were reported from food allergen-specific nanobodies empowering allergen-specific nanobodies as equivalent and serious agents compared to monoclonal IgG antibodies [44,45,46,47,48] (Table 1).

### 2.2. Importance of Affinity to Specific and Related Allergens

Affinity is a measure describing the magnitude of strength between the paratope of an antibody and an individual epitope of the antigen/allergen. It is expressed as the dissociation equilibrium constant K (K_D_), which is determined by the ratio of association (k_a_) and dissociation (k_d_) rate constants. While affinity measurements can be performed with a variety of different techniques, the kinetic parameters can only be determined by real-time evaluation [78]. Precisely, the speed and duration of the molecular interaction with an allergen are essential for the successful development of nanobody-based therapeutics. So far, several high-affine nanobodies have been described, including pollen and food allergen-specific nanobodies, but also IgE- and cytokine-specific nanobodies [44,46,48,50,79,80]. For nanobodies specific for Bet v 1, slow dissociation rate constants in the range of 10^−4^/s and 10^−5^/s were reported [44,45]. These stable complex bindings were even above the SPR-measured dissociation rate constants between 10^−3^/s and 10^−4^/s of Bet v 1/monoclonal antibody complexes, which have already proven to be effective in clinical studies [27,28]. Furthermore, an IgE-specific nanobody with a K_D_ = 1.4 nM revealed to bind free IgE with a higher potency than Omalizumab, an observation exemplifying the importance of the high affinity of biologics useful for allergy treatment [79]. Highly affine nanobodies were also shown to be reliable detection reagents for the accurate determination of major food allergen concentrations in food matrices facilitating a comprehensive analysis in the complete food chain [81] (Table 1).

### 2.3. Importance of Epitope Specificity and the Potential to Block the IgE–Allergen Binding

Determination of epitope specificity is a critical component in characterizing allergen-specific nanobodies as this feature determines the inhibitory potential to block IgE binding and IgE-mediated reactions such as basophil activation [44,49,52,72]. Methodologies for epitope mapping range from simple ELISA-based assays, where linear peptides are used, to more complex experimental techniques such as mass spectrometry, X-ray crystallography, and nuclear magnetic resonance (NMR) [82,83]. As it was postulated that nanobodies basically recognize conformation and prefer concave epitopes [54], classical ELISA based on linear peptide binding turned out to be inadequate to screen generated allergen-specific nanobodies for their epitope specificity. Since the epitope localization of nanobodies was only a surrogate to determine the IgE blocking capacity of generated nanobodies, we have applied ELISA inhibition assays to evaluate the competitive allergen binding of nanobodies and serum IgE antibodies. Using this inhibition approach and further allergen-induced basophil activation tests, we succeeded to identify Bet v 1-specific nanobodies that recognize a prominent IgE epitope and hence comprise the potential to reduce the mediator release of effector cells [44] (Table 1). Trimeric Bet v 1-specific nanobodies had an even superior blocking effect on basophil degranulation induced by cross-reactive allergens from pollen and foods. This finding certified that nanobodies can be easily and specifically tailored to increase cross-protection [45]. Epitope specificity was also crucial for the aforementioned anti-IgE nanobody that is able to displace IgE from FcεRI, which led to the inhibition of allergen-mediated basophil activation [79].

## 3. Diverse Application of Nanobodies in Allergy Research and Treatment

### 3.1. Nanobodies as Tools for the Determination of Allergen Concentration in Food, Air Samples, and Crude Allergen Extracts

**Figure 1:**
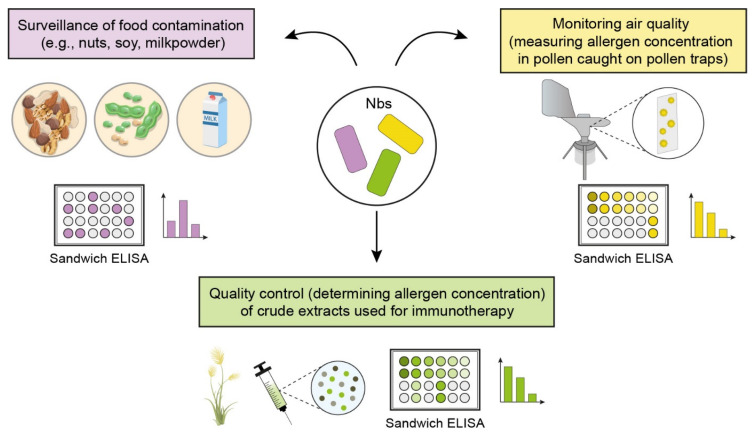
Versatile applications of allergen-specific nanobodies for allergen quantification. Nanobody-based immunoassays are already employed for food allergen inspection but are currently also developed to monitor allergenic load in air samples and might be helpful for quality control of crude extracts used for immunotherapy.

For several years, food safety has been a hot topic, in particular for allergic people, and has demanded the development of reliable and efficient test systems to carefully examine the production pipeline and final food products. Above all, contamination with nuts represents a serious problem for affected individuals, causing severe allergic reactions like anaphylactic shock. To ensure consumer safety, nuts have to be indicated as ingredients on packages to prevent unintended consumption. Additionally, nut-free labeled products need to be accurately checked for traces of cross-contamination by nuts. For these purposes, different methods are available, including fast, qualitative tests like rapid allergen detection strips, or more time-consuming but quantitative and highly sensitive immunoassays as well as real-time PCR [84]. Besides the aforementioned detection techniques, nanobody-based immunoassays have found their way as powerful tools into food analysis and strengthened their sentinel role at the front line [81]. Their potential to identify epitopes that are inaccessible for monoclonal antibodies recently facilitated their development for such applications. Aggravated by the popularity of vegan food, often enriched with nuts or lupine to increase the protein content, or due to feeding deeply hydrolyzed milk powder to milk-allergen-sensitized children, allergic reactions caused by food allergens are on the rise and represent a major issue. Therefore, nanobodies specific for the major allergens from peanut, macadamia nut, milk, and lupine have been validated lately to monitor allergenic components in different foods [46,47,48,50,51,85] (Table 2, Figure 1). Recently, nanobodies specific for tropomyosin, one of the most clinically relevant allergens in crustaceans, have been generated and successfully employed for precise food screening [86,87] (Table 2). Furthermore, nanobody-based immunoassays have already been successfully applied to uncover foodborne pathogens like mycotoxins in rice grains or bacteria (*Staphylococcus*, *Salmonella*, or *Listeria*) in milk samples posing a serious threat to public safety [88,89,90,91]. For instance, nanobodies showed superior efficacy over monoclonal antibodies due to the lack of the Fc region, which could otherwise mistakenly bind to *Staphylococcus* and cause false-positive results [89].

Similar to allergen management for food safety, there is an urgent need for the solid determination of allergenic load in our environment. To date, ambient air samples are solely characterized according to the pollen number and pollen phenotype. These observations give an idea about the composition of pollen grains but offer no precise amount of allergen concentrations released by atmospheric pollen. However, it is well accepted that the relation between airborne pollen counts and pollen allergen levels is unpredictable [100,101]. The quantity of allergens released from pollen grains varies significantly depending on the geographic location, seasonal time, and on meteorological factors [102]. Furthermore, it has been extensively described that certain parameters like CO_2_, NO_2,_ and Ozone concentration influence the expression of allergens in plants, provoking variable allergenic potential [103,104,105,106]. A recent panel study showed for the first time that the quantity of Phl p 5, a major allergen from grass pollen, but not pollen counts, is consistently associated with allergic respiratory symptoms [100]. To determine Phl p 5 concentrations in the sampled air, the authors relied on an antibody-based immunoassay. This pioneering study reveals the long sought-after association between allergen exposure and allergic complaints. It will further pave the way for linking the intensity of allergic reactions with the available levels of aeroallergens from other pollen sources, but also from mold spores and dust samples, both allergen sources that have been associated with severe respiratory exacerbations. Based on the mutability of already-generated allergen-specific nanobodies, it should be easy to expand the current repertoire of nanobodies to establish nanobody-based approaches to monitor outdoor and indoor allergen loads [44,93] (Figure 1). Such air quality data shall represent a must-have in the near future and a prerequisite for serious local allergen forecasts.

First efforts have already been made to build up platforms using social media to disseminate information on pollen levels but also to gain knowledge about regional climatic influence on pollen counts to develop allergy risk prediction models and tailored awareness campaigns [107]. In the future, such services could be expanded by including data of allergen concentrations.

While the quality control of complex allergen extracts for diagnostic purpose lost its importance with the implementation of diverse microarray-based tests (e.g., ISAC, ALEX, FABER) [108,109,110], the precise determination of concentrations of single allergens in crude extracts that are still widely used for AIT is a long-cherished goal [11,15]. The knowledge that certain extracts either lack important allergens or differ in their ratio of single allergens and/or can be contaminated with molds [111,112,113] sparks a third application area of allergen-specific nanobodies as an evaluation tool for allergen extracts. Reliable test systems based on validated allergen-specific nanobody pairs could be set up quickly to examine pollen extract compositions and ultimately determine if the marketed aqueous extracts contain enough allergen quantities to ensure clinical effectiveness (Figure 1).

### 3.2. Passive Immunization with Allergen-Specific Nanobodies

Passive immunization has a long history in allergies and has recently experienced a renaissance. As early as 1911, Leonard Noon was one of the first to describe the generation of immune tolerance against a corresponding allergen following the subcutaneous injection of pollen extracts. At this time, the principle behind this procedure, which is currently known and applied as AIT, was still a mystery [114]. Several decades later, studies to uncover underlying protective effects were performed, indicating the existence of an inhibiting substance within the sera of sensitized patients treated with AIT that prevents the interaction between the allergen and the sensitized cells [115]. In 1940, Mary H. Loveless confirmed these results by identifying a special antibody type as the inhibiting substance. This thermostable antibody binds to the very same allergen as the sensitizing antibody and was later designated as IgG. She further stated a dependence of the protective effect on the amount of blocking antibodies [116]. The concept of passive immunization was born by demonstrating the successful prevention of in vitro histamine release after the pre-incubation of IgG antibodies with allergens, proving the inhibition capacity of these antibodies [117]. Since then, several studies used the idea of directly injecting blocking IgG antibodies with immediate effect instead of applying a time-consuming AIT. For instance, antibodies raised against the major grass pollen allergens Phl p 1 and Phl p 5 and the birch pollen allergen Bet v 1 showed the effective inhibition of basophil degranulation with a protective effect lasting up to three weeks in grass- and birch pollen-sensitized mice, respectively [118]. A very recent study uncovered blocking IgG antibodies able to prevent anaphylaxis in mice induced by the peanut allergen Ara h 2 [119]. A real game changer in the field of passive immunization for treating allergies has been the development of two monoclonal antibody cocktails which have already succeeded in their first clinical trials. The first antibody mix (REGN1908-1909) was designed to target the major cat allergen Fel d 1 by binding two distinct IgE epitopes on the allergen surface (Table 1). This convincing novel treatment approach reduced in vivo symptoms such as labored breathing ability and asthma reactions for up to three months and proceeded to a phase III clinical study [29,30,120,121] (https://www.clinicaltrials.gov (accessed on 2 July 2024), NCT04981717). Unfortunately, this phase III trial has very recently been terminated due to lack of efficacy. The second very promising antibody mix deriving from the same platform consists of the monoclonal IgG antibodies REGN5713, REGN5714, and REGN5715 targeting three different IgE epitopes on the major birch pollen allergen Bet v 1. This combination of antibodies inhibited basophil degranulation in more than 90% of tested sensitized patient sera (Table 1). Importantly, this antibody melange demonstrated a reduction in allergic symptoms with a lasting effect of up to 2 months, thereby showing great potential for seasonal allergies, and is currently under investigation in a phase III clinical study (https://www.clinicaltrials.gov (accessed on 2 July 2024), NCT04709575) [27,28]. However, to justify the broad clinical applications of monoclonal antibodies for allergic rhinitis, comprehensive safety studies are ongoing to independently demonstrate their harmlessness [122].

Besides inhibiting effector cell degranulation, other features of IgG antibodies for passive immunization include interference with IgE-facilitated allergen presentation (FAP) and subsequent reduction in T cell activation [8,123,124,125]. When affiliated with an allergen, IgE binds stronger to CD23 expressed on several immune cells, e.g., B cells. This allergen–IgE–receptor complex is internalized, processed, and allergen peptides are then presented to specific T_H_2 cells [8]. It has been shown multiple times that monoclonal antibodies or AIT-induced blocking antibodies inhibited facilitated allergen binding (FAB) to B cells, which served as a surrogate model for FAP, leading to T cell activation [126,127,128,129]. Moreover, blocking antibodies might be involved in diminishing a secondary immune response (i.e., IgE production) which normally arises from the allergen-induced activation of memory B cells [130,131,132]. Long-term sensitization is achieved with memory B cells that give rise to the typical boost of IgE-producing cells after re-exposure to the corresponding allergen and its binding to the B cell receptor (BCR). Nevertheless, IgE memory B cells are extremely rare and probably play a minimal role in the extensive recall response, leading to the consideration that IgG memory B cells are also activated and undergo class switch recombination to IgE [133]. In any case, shielding the allergen from interacting with the BCR should interrupt cell activation, differentiation to plasma cells, and IgE production. It has been observed that after AIT, the expected allergen-specific IgE boost during pollen season was dampened; however, whether this consequence is attributed to blocking antibodies or the induction of anti-inflammatory cellular responses such as IL-10 or IL-35 production is not entirely clear yet [134,135,136,137].

Although blocking IgG antibodies seem to be very effective in inhibiting interactions between patients’ IgE antibodies and allergens, their production tends to be laborious and very expensive. As a result, more and more scientists focus on smaller antibody-derived fragments such as nanobodies. Nanobodies have already demonstrated comparable characteristics and, most importantly, inhibition capabilities as whole antibodies. We were the first to propose a treatment approach for allergies that is based on these small antibody fragments with the development of Bet v 1-specific nanobodies (Table 2). They exhibit high affinities to their cognate allergens and are moreover able to recognize cross-reactive allergens. They could not only convince with their affinities and cross-reactivities but especially with their ability to inhibit IgE–allergen interactions on basophils, thereby reducing mediator release [44]. These already impressive properties were even improved by generating a trimeric nanobody construct based on one of these nanobodies. This nanobody trimer exceeded cross-reactivities and was further able to strongly inhibit basophil degranulation induced by Bet v 1 as well as Aln g 1 and Cor a 1 [45]. So far, these blocking nanobodies have only been tested in in vitro settings, and in vivo studies are required to confirm their potential for passive immunization for allergic patients. Meanwhile, another group has produced nanobodies against honey bee venom and grass pollen allergens [92,93] (Table 2). Although they formulated IgE-like nanobodies for diagnostic purposes, they discussed the possibility of using their platform for developing IgG-like formats that can be applied in interventional studies. In the case of honey bee venom allergy, AIT is well established and effective, and passive immunization with nanobodies may not be suitable, especially since the occurrence of insect stings is usually unpredictable. However, seasonal pollen allergy is a reasonable opportunity for nanobody application, and their potential to overcome the issue of insufficiently induced cross-protection by AIT to pollen-related food allergens is a valid motivation for supporting continued research.

**Figure 2:**
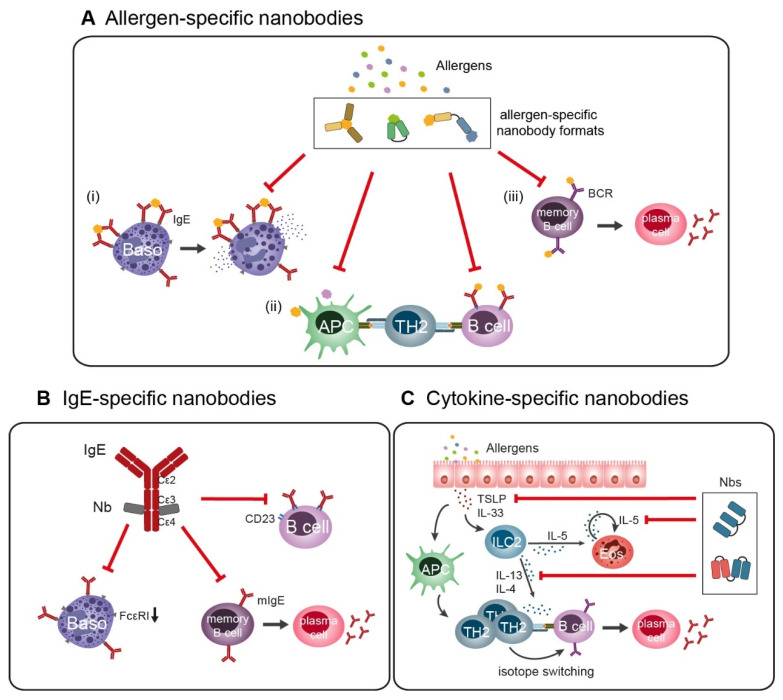
Strategies for developing nanobody-based formats for treating IgE-mediated allergic diseases. (**A**) Allergen-specific nanobodies (mono- or multivalent, multi-paratopic or multi-specific) can block allergen interactions with IgE, hence (i) reducing effector cell degranulation, (ii) interfering with the uptake of allergens by APCs, as well as IgE-facilitated allergen presentation to T cells, and (iii) blocking activation and differentiation of memory B cells. (**B**) Directly targeting IgE results in the displacement of bound IgE to its two receptors (FcεRI and CD23) and reduces overall serum IgE levels. Targeting IgE can also be used to trace membrane IgE-bearing (memory) B cells and downregulate IgE secretion by preventing differentiation to plasma cells. (**C**) Neutralizing certain cytokines interferes with the pathways of progression and maintenance of allergic diseases. TSLP, IL-5, and IL-13 are currently targeted by nanobodies, but other cytokines such as IL-4 and IL-33 may also represent attractive targets in the future. Abbreviations: APC, antigen-presenting cell; Baso, basophil; BCR, B cell receptor; Eos, eosinophil; ILC2, type 2 innate lymphoid cell; mIgE, membrane IgE; Nb, nanobody; T_H_2, T helper 2 cell; TSLP, thymic stromal lymphopoietin.

First attempts have already proven nanobodies’ ability to block FAB to B cells as a consequence of shielding IgE epitopes [45]. However, it remains to be investigated whether nanobodies are able to (i) prevent FAP by B cells, (ii) hence suppress T cell activation and (iii) hamper the activation of memory B cells to differentiate to IgE-producing plasma cells (Figure 2A).

### 3.3. Targeting IgE and Type 2 Cytokines

The allergic reaction involves many more molecules than just the allergen that can be targeted with biologics. IgE represents a key driver in the biochemical cascade that was identified as a critical component decades ago, which, when neutralized, leads to improvement in clinical symptoms [138,139,140,141]. In fact, the development of the IgE-specific monoclonal antibody Omalizumab was a pioneer for managing allergies [142,143,144]. It is approved for the treatment of moderate-to-severe allergic asthma [145] and chronic spontaneous urticaria [146], and it is also under investigation for treating allergic rhinitis [147]. Omalizumab binds to the Cε3 domain of IgE, thereby inhibiting the binding of free serum IgE to its receptor FcεRI [148], which in turn downregulates overall receptor expression on mast cells and basophils [149,150]. A potential successor to Omalizumab is Ligelizumab, a next-generation high-affinity IgE-binder recognizing an epitope that overlaps with the binding site to FcεRI, thus strongly reducing mast cell degranulation. It is, therefore, a promising candidate to be tested in clinical studies for treating allergic diseases [151,152].

Besides humanized anti-IgE antibodies, polyclonal camel HCAbs against IgE have been isolated that block histamine release of human basophils [94]. When producing a recombinant anti-IgE HCAb version deriving from the PBMCs of the same camel, the authors failed to generate highly specific HCAbs, and hence their enthusiasm to develop orally administered immunotherapeutic agents faded [95] (Table 2). The generation of a llama-derived anti-IgE nanobody revived their efforts [96]. This nanobody was the first assigned to the class of disruptive IgE inhibitors due to its mode of action [79,97] (Table 2). In a bispecific format (designated ALX-0962) additionally targeting human serum albumin to prolong its plasma half-life, it was shown to bind free IgE with a higher potency than Omalizumab. More importantly, ALX-0962 was able to displace FcεRI-bound IgE from basophils in in vitro assays, resulting in lower degranulation compared to Omalizumab treatment [97]. Detailed structural analysis of the IgE-binding nanobody (also known as 026 sdab) revealed that it does not bind to the FcεRI epitope (Cε3) but to the region between Cε3 and Cε4. This locks the IgE-Fc in a bent, closed conformation, which does not allow for an interaction with FcεRI. Importantly, this structural change also disrupts already formed IgE:FcεRI complexes. Furthermore, the binding epitope of the nanobody is overlapping with the binding site of CD23, which results in the inhibition of binding to this receptor as well [79] (Figure 2B). Further development of the nanobody was unfortunately discontinued, but, nevertheless, the remarkable mode of action may open up the doors for developing novel anti-IgE drugs that target allosteric sites, thus inducing conformational changes.

Blocking IgE might also lead to the suppression of IgE bearing (memory) B cells (Figure 2B). A study brought forward the first evidence that a single-chain anti-IgE fragment was able to induce a tolerogenic signal to IgE+ B cells by binding to membrane IgE (mIgE). The overall IgE expression, development of IgE plasma cells, and IgE secretion were found to be decreased in anti-IgE-treated sensitized mice. This antibody also had the capacity to neutralize serum IgE and detach IgE from mast cells and basophils [153]. However, no continual project elaborating such pioneering findings has been published so far, whereas the literature describing high-affinity monoclonal anti-IgE antibodies that eliminate mIgE-expressing cells via antibody-dependent cell-mediated cytotoxicity is broad [140,154,155]. For instance, it was shown that Fc portions of IgE-specific IgG antibodies could be mutated to enhance their affinity for the IgG receptor FcγRIIIa, which plays a role in mediating effector functions [156]. Accordingly, it was assumed that B cells are eliminated and IgE serum levels are diminished. While the short in vivo half-life of the first applied IgE-specific IgG antibodies enabling only a temporary beneficial effect had to be stopped after phase I [157], new IgE-specific antibodies that efficiently downregulate CD23-mediated IgE synthesis are on the rise and will also pave the way for streamlined nanobody evolution [158].

Obviously, nanobodies alone cannot orchestrate these functions due to the lack of Fc, but IgG formats of nanobodies may easily be generated. These examples solidify the far-reaching effects of anti-IgE treatment in allergies and demonstrate the exciting opportunities that nanobody development could achieve.

Besides IgE, type 2 cytokines like interleukin (IL)-4, IL-5, and IL-13, or inflammatory epithelial cell-derived cytokines like thymic stromal lymphopoietin (TSLP) or IL-33, can also be successfully targeted to ameliorate allergic symptoms, since they are critical components in the initiation, progression, and maintenance of allergic diseases (Figure 2C). IL-4 plays an important role in T_H_2 differentiation and together with IL-13 induces antibody isotype switching to IgE in activated B cells [159]. It has been shown that blocking their shared receptor IL-4Rα with a monoclonal antibody can reduce type 2 inflammatory markers and improve symptoms in allergic rhinitis and comorbid asthma or allergic dermatitis [160,161,162]. Recently, a multimeric, directly IL-13-targeting nanobody that prevents the cytokine from binding to its receptor IL-13Rα has been described [80] (Table 2). Multiple generated nanobodies binding to different epitopes with high affinity were connected to bi- and trimeric formats to enhance their biological inhibitory potency. Although this study only investigated the inhibition of the receptor, and further experiments to establish clinical efficacy have yet to be performed, the authors claim that their nanobody-based construct might be used as the basis for treating IL-13-related diseases, with a particular focus on asthma. This is of certain interest since the authors noted that most monoclonal antibodies targeting IL-13 showed limited efficacy in clinical trials treating asthma. They questioned the subcutaneous or intravenous administration routes in these studies and proposed the pulmonary route for a local application, where nanobodies might end up being advantageous over full antibodies due to their stability [80]. Taking it a step further, a recently completed phase I clinical trial investigated the potential of the dual blockage of IL-13 and TSLP in asthma via a bifunctional nanobody format (https://www.clinicaltrials.gov (accessed on 2 July 2024), NCT05366764) (Table 2). In this randomized, double-blind, placebo-controlled study, a single dose of the nanobody-based drug (SAR443765) was administered subcutaneously to 36 participants with mild-to-moderate asthma. The primary endpoint was safety, and the secondary endpoint was a change in fractional exhaled nitric oxide (FeNO), a recognized biomarker for respiratory inflammation, in comparison to placebo over four weeks after the treatment. The full outcome of this trial has yet to be published, but the preliminary results show that the treatment was well tolerated and FeNO levels were significantly reduced as early as week 1 in the treatment group compared to the placebo group. Type 2 blood biomarkers such as IgE, IL-5, and eosinophils were also lowered [98]. Accordingly, a following phase IIb trial investigating efficacy, safety, and tolerability with dose ranging has just been initiated (https://www.clinicaltrials.gov (accessed on 2 July 2024), NCT06102005).

Another inflammatory cytokine, IL-5, is an attractive target in eosinophilic asthma. IL-5 enhances mucus production and recruits eosinophils to the inflamed tissue site, where they drive tissue remodeling. Furthermore, eosinophils themselves produce high amounts of IL-5 critical for their differentiation, proliferation, and function [163]. To disrupt this positive feedback loop, researchers have developed a trivalent bispecific nanobody with two IL-5-binding domains, notably to two different epitopes, and one albumin-binding domain (IL-5-HSA Nb) [99] (Table 2). Compared with an already approved monoclonal antibody against IL-5 (mepolizumab), this nanobody construct was superior in its potency to block receptor binding and the proliferation of TF-1 cells, a model system for eosinophils. Concluding in primate studies, IL-5-HSA Nb showed sustained pharmacokinetics (half-life time: 12 days) owing to its albumin-binding domain, and significantly suppressed blood eosinophil levels for two months, reaching normal levels after approximately 84 days [99]. This makes it an interesting next-generation therapeutic for treating eosinophilic asthma. Notably, its clinical efficacy has not yet been investigated in disease models; however, its convincing pharmacokinetics and pharmacodynamics could propel its development to clinical studies in humans.

Taken together, targeting the allergic pathway and the more general aspects of inflammation is beneficial for patients that experience severe symptoms such as asthma. Often, this correlates with sensitization to multiple allergens [164], in which case eliminating one allergen alone is not sufficient to alleviate symptoms. Monoclonal antibodies are paving the way, but the robust, cheap, and easy-to-manipulate nanobody formats might be advantageous, as seen in the currently developed examples.

## 4. Challenges and Perspectives

### 4.1. Half-Life of Nanobodies

One of the largest limitations for nanobodies as therapeutics is their generally short half-life. For several reasons, e.g., as a molecular probe for cancer imaging, a short lifespan in the body is desired and suffice to serve the purpose. However, when it comes to treatment, a prolonged half-life is definitely required. For instance, passive immunization approaches for pollen allergy utilizing IgG antibodies have already demonstrated that half-life times of several weeks are needed to provide protection throughout the pollen season [28] (Table 1). In general, extended drug availability in the circulation reduces medication intervals, regardless of seasonal or non-seasonal allergies, emphasizing that a long half-life is most valuable. The half-life time of proteins varies a lot and is mostly ascertained through the determination of the renal clearance, which depends on the permeability of the glomerular membrane [165]. The glomerular permeability of a protein is largely characterized by two key features, which are the dimension (directly proportional with the molecular weight) and the charge of a molecule [166,167]. It was shown that molecules smaller than 20 kDa were quickly eliminated through the kidneys, while molecules larger than 60–70 kDa were hardly present in the glomerular filtrate. These findings indicate that substances with a higher molecular weight show a low degree of renal clearance [165,166]. However, the size of a molecule alone is not sufficient to determine the glomerular permeability. The importance of the charge of a molecule was proven to be decisive for its elimination. Positively charged dextran molecules displayed a much higher degree of clearance than neutral or negatively charged ones, making the charge a crucial consideration for estimating the in vivo half-life [167]. Hence, the molecular weight and the charge can not only help to predict half-life times but can be used to engineer the isoelectric point of antibodies accordingly to define their duration in the human body. Representative studies have shown that selected amino acid exchanges in the framework or the variable region of therapeutic antibodies, resulting in a lower isoelectric point, led to decreased blood clearance [168,169].

Owing to their small size, nanobodies are rapidly cleared by the kidneys. Although they can achieve quite high affinities to their cognate antigens, their serum half-life times are limited to a few hours, a fact that hampers their efficacy for allergy treatment [170,171]. To achieve clinical usage, nanobodies must persist much longer in the blood circulation and body depending on their area of application. Various methods have been developed over the past decades to engineer proteins/nanobodies in order to increase their abundance in circulation. These methods can be roughly divided into three sections: (i) increase in the molecule’s dimension (dimerization, multimerization), (ii) fusion or direct binding to negatively charged structures like polyethylene glycol (PEG), and (iii) utilization of the neonatal Fc receptor (FcRn)-mediated recycling pathway [55] (Table 1).

The bivalent nanobody Caplacizumab, when bound to its target, exhibits a terminal half-life of 17–30 h in animal models [172] and 9–60 h in humans depending on the mode of administration [173,174], despite its small size of approximately 28 kDa. Studies showed that Caplacizumab bound to vWF is excreted via hepatic clearance and follows a different route of elimination than unbound nanobody, which is quickly secreted by the kidneys [172,173]. However, its short half-life implies injections on a daily basis, a fact that makes Caplacizumab an expensive drug and has started an ongoing discussion on the cost-effectiveness of the first approved nanobody [65]. Pharmacokinetic studies from another bivalent nanobody, targeting VEGF, revealed a terminal half-life of 90 min in mice [175]. These results demonstrate that besides the size of injected protein drugs, other factors such as the species or the presence of target-bound or unbound nanobodies influence their persistence and therefore their in vivo half-life. Consequently, dimerization is generally not sufficient to drastically prolong half-life time in vivo [176].

To increase the molecular weight of a protein and therefore its dimension beyond nanobody dimers, multimerization can be an easy solution. We and others have pointed out that post-translational trimerization of nanobody monomers by introducing trimerizing domains (e.g., isoleucine zippers, human collagen XVIII) can increase the molecular weight to approximately 70 kDa [45,177,178,179]. Trimerization can further enhance biological activity compared to the monomeric form. Increased virus neutralization capacity by trimeric nanobodies was demonstrated [177,178], which might be translated to treating allergies as well. As mentioned above, blocking allergens from IgE binding reduces IgE-mediated effector cell activation and downstream signaling, and a first study has shown that allergen-specific nanobody trimers performed in a superior manner to their monomer counterparts [45]. Besides improved bioactivity, renal clearance and therefore early elimination might be avoided with such approaches, but in vivo confirmation is required. Trimerization can not only be achieved by adding trimerizing domains but can already be engineered at DNA level through the linkage of nanobody monomers via linker sequences such as the common Gly–Ser linker [180,181].

A popular alternative to trimerization is the fusion to PEG, referred to as PEGylation. This well-established method entails the increase in the hydrodynamic size and the masking of positive charges of proteins, leading to a delay of elimination [182]. Recent studies investigating the in vivo half-life of PEGylated nanobodies compared to the corresponding parental unconjugated nanobodies verified this statement by demonstrating prolonged presence in blood circulation in different animals [183,184]. However, since PEG is non-biodegradable, intralysosomal accumulation is assumed [185]. Furthermore, although rare, intolerance or allergic reactions to PEG have become evident in the past years [186]. An alternative could be conjugation with polysialic acids (PSAs). PSAs do not only increase the dimension of a protein but contribute to the impairment of renal clearance with their high ionic state [185,187].

One of the most favored approaches to extend half-life is to make use of the already existing recycling pathway mediated by FcRn. Endocytosed albumin and IgG are captured in acidic compartments, recycled back to the cell surface, and released after dissociation at neutral pH. This recycling system is responsible for the unusually long half-life of about three weeks for albumin as well as IgG [56,188].

The most straightforward way of hijacking this pathway is the direct binding to albumin [56]. Different albumin-based approaches have been developed, including direct fusion to albumin as well as the generation of multi-specific nanobodies binding to albumin and the antigen(s) of choice. In this context, it was already shown that albumin-specific nanobodies reached half-life of several days in animal models [170,189,190]. The first Japan-approved trivalent nanobody based on this approach is Ozoralizumab, with an impressive in vivo half-life time of 18 days, evidencing the functionality of albumin-binding nanobodies [191,192,193]. Another method that was already applied for nanobodies is the fusion to the Fc region of an IgG antibody [194,195], which would not only increase the half-life time but can also mediate IgG effector functions, e.g., the activation of anti-inflammatory pathways in the context of allergies [8].

Very recently, different application routes were reviewed and shown to have an impact on the longevity of antibody pharmaceuticals [55]. The gained knowledge will certainly support taking all the necessary measures to optimize nanobodies’ delivery system to achieve long-acting drugs.

To conclude, multiple strategies are available to improve the half-life of therapeutical nanobodies. Since these techniques have already been proven useful in different diseases, we anticipate that with the rise of interest in nanobody-based applications in allergies, more studies concerning appropriate nanobody formats for allergy treatment will be published in the near future.

### 4.2. Immunogenicity and Humanization Strategies

While it is generally claimed that nanobodies possess a low immunogenicity risk profile due to their high homology with the human IGHV3 gene family, peer reviewed publications referring to immunogenicity data of nanobodies are surprisingly rare [57,59,196] (Table 1). However, it is common knowledge that preclinical safety testing of all novel biopharmaceuticals, including nanobodies, is required to select the most appropriate candidate for further clinical development. Integral parts of preclinical safety testing are the detection of (pre-existing) anti-drug antibodies (ADA), evaluation of aggregation propensity of the prospective agent, and the capacity to stimulate key players in the immune reaction. Therefore, panels of human serum samples, in silico analyses to predict potential T and B cell epitopes or T-cell-based stimulation assays are applied to evaluate the probability of adverse events. Dynamic light scattering, affinity-capture self-interaction spectroscopy, or similar methods support the elucidation of the size and shape of molecules and offer useful information about the homogeneity of proteins and their tendency to form aggregates. Finally, to translate in vitro findings to clinical development, the immunogenicity potential has to be examined in experimental animal models. Based on the finding that even nanobodies that do not react with murine antigens are immunoreactive with human antigens indicated non-human primates as a more reliable model to mirror clinical outcome [57,197]. Luckily, tissue- and cancer-derived organoid research continued to gain ground and was recently adopted to the field of allergies. This cutting-edge technology will offer a reasonable and accurate alternative to animal testing in the near future.

To date, we have found only a few manuscripts reporting on the preclinical immunogenicity risk potential of nanobodies and their humanized derivatives. One study focused on two non-humanized nanobody monomers applied for PET imaging. Both nanobodies did not form aggregates, and neither did they activate dendritic cells nor induce T cell proliferation. Importantly, the authors could show that the applied nanobodies neither induced anti-drug antibodies (ADAs) nor did they enhance pre-existing ADAs which were found in only low amounts in 1 out of 20 breast carcinoma patients. Based on these observations, the authors concluded that the administration of these nanobodies has no negative effect on the clinical outcome and paved the way for a clinical phase II study [196]. Although repeated doses of nanobodies administered during a phase II study could favor the generation of ADA, patients did not show adverse reactions. These encouraging results foster the further clinical development of these nanobodies as PET tracers in breast cancer patients [198]. Confirmation comes from a recently published paper pursuing the same issue and found that repeated injections of a non-humanized EGFR-specific nanobody monomer in healthy dogs is well tolerated [199]. Still, earlier reports exist that refer to the discontinuation of nanobody studies due to the presence of pre-existing ADAs in patients [57,200,201]. The authors observed clinical and physiological signs of cytokine release in few subjects with pre-existing ADA after injection of nanobody monomers [200]. The same nanobody monomers did not elicit any symptoms when administered via inhalation in a clinical trial involving healthy human subjects [202]. These controversial outcomes point to the significance of immunogenicity investigations as a critical step during therapeutic nanobody development. A comprehensive overview about the preclinical and clinical immunogenicity assessments of humanized and non-humanized single-domain antibodies was recently given, and essential information can be found there [57] (Table 1). The lessons learned from these published nanobody safety studies outside the field of allergies will certainly drive the progress in the development of safe nanobody vaccines for allergy treatment forward and will minimize time-consuming animal testing. Furthermore, the recent implementation of deep learning tools enabling the precise prediction of the nativeness of Fv sequences of antibodies and nanobodies and hence of the likelihood of immunogenicity provides a rapid way to even overcome animal testing [58]. Relying on these accurate predictions that make use of the volume of available sequences and structural data, programs like “AbNativ” or “Llamanade”, both user-friendly, open-source computational pipelines, are also able to suggest rational humanization. This intriguing achievement will definitely support the replacement of elaborating strategies developed in the last 30 years, including de-immunization and resurfacing [58,203]. Such programs provide a holistic approach to select nanobody equivalents (comparable to native nanobodies derived from the immune system) comprising low self-antigen cross-reactivity, low immunogenicity, and beneficial half-life. The forementioned characteristics are decisive to increase human compatibility and to eventually foster successful clinical development [58].

### 4.3. Local Allergy Treatment—A Glance into the Future

**Figure 3:**
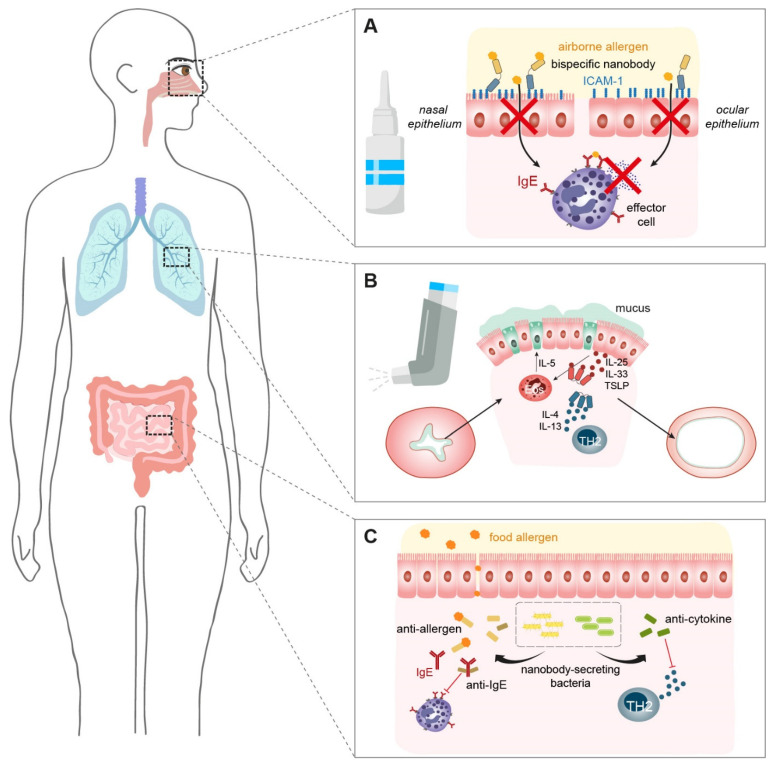
Prospective nanobody-based treatment options for allergies. (**A**) Nanobodies bispecific for allergens and ICAM-1 applied via a nasal spray or eye drops may provide a protective shield against allergen entry. (**B**) Nebulized nanobodies retain their biological activity and can be used in inhalers. Applying nanobodies against carefully selected targets (e.g., IL-5, IL-13, TSLP) may alleviate bronchial restriction in asthma patients. (**C**) An innovative drug delivery approach suggests administering engineered symbiotic bacteria to colonize the gut that secret functional nanobodies directly into their surroundings. In terms of food allergies, this model might be able to deliver, e.g., allergen-, IgE- or cytokine-specific nanobodies to the local inflammation site to treat allergic symptoms.

Our review shows that there is a lot of potential for the application of nanobodies in allergen surveillance as well as allergy treatment. So far, we have only introduced therapeutic concepts that work systemically, but we also want to point out the option of local administration. This delivery path offers the advantage of a quicker onset of therapeutic effects and might also avoid possible adverse events. For respiratory allergies, the nose as an administration route for allergen-specific antibodies has been proposed recently [204]. We have developed a nanobody recognizing intercellular adhesion molecule-1 (ICAM-1) [205], a cell surface receptor that is highly upregulated at the apical surface in nasal epithelial cells in allergic patients [206]. We proposed that by linking the ICAM-1-specific nanobody to a second, allergen-specific nanobody, this format is able to catch allergens at the entry point and prevent subsequent allergen-induced symptoms. This idea has been investigated in two proof-of-concept studies using monoclonal antibody conjugates [207,208]. These bispecific conjugates strongly reduced allergen penetration through a monolayer of human bronchial epithelial cells for up to 72 h and thus elicited significantly decreased release when tested with cultured rat basophilic leukemia cells. Importantly, it turned out that high-affinity binding to allergens suffices to capture allergens, while blocking the IgE–allergen interaction is not essential to efficiently act as a biological shield [208]. One downside of these studies is that chemically conjugated antibodies usually suffer from batch-to-batch inequalities. To further test this concept in animal models, a reliable composition of bispecific antibody-based constructs is needed. Nanobodies live up to this challenge by their modular nature and ease of formulation and production. We anticipate that we will see this concept being developed further by utilizing bispecific nanobodies. In a finalized form, these therapeutics could then be delivered to the mucosal lining via nasal pump sprays or special devices creating “pulsating aerosols”, with the latter resulting in a potentially prolonged deposition of the drug and additional localization in the sinuses [209]. Under inflammatory conditions, ICAM-1 is also upregulated in ocular epithelial cells [210]. Additionally, a clear connection between ocular surface and nasal epithelia is evident [210,211]. Based on their connectivity and since allergic rhinitis is frequently associated with comorbid allergic conjunctivitis (i.e., rhinoconjunctivitis) [212,213], it makes sense to also envision ICAM-1/allergen-specific nanobodies formulated as eye drops in the future (Figure 3).

For lower respiratory tract symptoms (e.g., asthma), inhalers are suitable tools to deliver therapeutics deep into the lungs. Due to their size and stability, nanobodies are able to withstand the sheer forces during the nebulization process [214]. Nebulized nanobodies have already been tested in respiratory viral infections. A nanobody targeting respiratory syncytial virus (RSV) retained its antiviral properties in rat studies and a clinical trial for hospitalized children [180,215]. During the SARS-CoV-2 pandemic, multiple groups developed anti-spike protein nanobodies that were stable as an aerosol and successfully neutralized viral titers in cultured cells [216,217] or in the lungs of infected animals via the inhalation route [218]. Noteworthy, the nebulization process did not affect their functionality in either the monomeric or the trimeric form [180,216], a great advantage since trimeric nanobody formats often achieve a more potent bioactivity. Furthermore, depositing the therapeutic nanobody directly into the lungs via an inhaler required three times less the amount needed to neutralize the virus compared to the intranasal route in the animal study [218]. Similarly, nanobodies that target the T_H_2 pathway (e.g., cytokines such as IL-5 and IL-13) could be delivered to the lungs to treat allergic asthma (Figure 3).

A smart method to deliver therapeutic nanobodies directly to the gut is by modifying commensal bacteria to secrete them into their surroundings. In mouse models of colitis, TNF-α-neutralizing nanobodies produced by orally administered *Lactococcus lactis* or *Escherichia coli* reduced intestinal inflammation and histopathological markers [219,220,221]. We can speculate that this kind of platform could also be applied in the context of food allergy. The interaction of food allergens with allergen-specific IgE-loaded effector cells in the gut can lead to massive inflammation in allergic individuals [222]. By secreting food allergen-specific, IgE-specific, or cytokine-neutralizing nanobodies directly to the inflamed sites, allergic reactions might be alleviated (Figure 3).

Since allergens usually enter the body through mucosal surfaces, strengthening mucosal immunity is also a valid option to treat allergic patients. The predominant antibody class at these sites is IgA, which has been recognized to exert a certain protective effect in the context of allergies [8,223]. In fact, during sublingual grass pollen AIT, allergen-specific IgA antibodies are induced, besides IgG_1_ and IgG_4_, and are thought to contribute to clinical improvements [224]. On the contrary, a recent study found no correlation between the abundance of gut allergen-specific IgA and protection or tolerance in the case of peanut and egg white allergy [225]. Nevertheless, it has been shown that monoclonal IgA antibodies are able to block IgE–allergen interaction and effector cell activation, and therefore might offer an alternative or additional option to IgG for passive immunization [226,227]. Nanobodies can be easily formatted as IgA by linking the corresponding Fc portion to the C-terminus and retain their cost-effective and scalable production in simple microorganisms such as yeast. A few nanobody–IgA constructs have already been applied for combating viral respiratory infections [228] or bacterial gastrointestinal infections [229,230] and even proven superior to their IgG counterpart in regard to their biological activity at mucosal surfaces [228]. Whether allergen-specific IgA nanobody formats have a similar advantage over IgG antibodies remains to be investigated.

## 5. Conclusions

Nanobodies are powerful and versatile tools that have become increasingly appealing in allergy research over the last decade. Many potential targets have already been identified for this class of biologics to intervene with the allergic reaction, including the interaction with the respective allergen, with IgE and with type 2 cytokines. Their beneficial qualities such as their high affinity to a large variety of epitopes, their chemical stability, their adaptability, and their inexpensive generation and validation distinguish nanobodies for their utilization in a wide range of applications.

While the majority of current studies related to allergen-specific nanobodies focus on allergen detection and quantification of potentially harmful allergen contamination in food matrices, only a few reports exist showing their therapeutic potential in inhibiting IgE binding and reducing basophil activation in vitro. On the other hand, there is considerable progress in targeting broader mechanisms with IgE- and cytokine-specific nanobodies. Notably, some of these compounds have successfully passed phase I clinical trials, and we are excited to closely follow their further evolvement. Published findings of these safety assessments will definitely accelerate the overcoming of identified challenges that need to be addressed for the successful development of nanobody-based therapeutics for allergy treatment. The short half-life continues to be an issue and represents a critical factor for the discontinuation of promising drug candidates after phase I clinical trials. Also, nanobody-induced immunogenicity is a fundamental topic to be considered. While current studies highlight that nanobodies are generally well tolerated, the occurrence of pre-existing ADAs has to be kept in mind when elaborating on efficient nanobody-based drugs. Since the appearance of the first report on allergen-specific nanobodies in 2019, a lot of knowledge about these tiny powerhouses has been collected, proving unambiguously that nanobodies are on the road to combatting allergies.

## Figures and Tables

**Table 1:** Characteristics of allergen-specific nanobodies and monoclonal antibodies.

	Nanobody	Conventional Antibody (IgG)
Specificity	Highly specific [44,45,46,47,48]	Highly specific [27,49]
Cross-reactivity	Broad cross-reactivity to related allergens [44,45,46,47,48]	Broad cross-reactivity to related allergens [27,49]
Affinity	In the range of K_D_ = 10^−6^ to 10^−10^ M [44,46,48,50,51]	In the range of K_D_ = 10^−6^ to 10^−11^ M [27,29,52,53]
Epitopes	Rigid, structured, concave epitopes [54]	Linear peptides, flat or convex surfaces [54]
IgE blocking potential	Effective blocking potential, but more than one nanobody is needed for full IgE blocking [44,45]	Effective blocking potential, at least two antibodies are needed for full IgE blocking [27,29]
Half-life in vivo	A few hours at most for monomeric nanobodies; can be increased by larger constructs or fusion to albumin/Fc/PEG [55]	Up to three weeks due to recycling via the neonatal receptor (FcRn) [56]
Immunogenicity	Due to a high similarity to human VH, VHHs are considered low immunogenic [57] but need to be evaluated individually	(Non)-human antibodies can lead to the induction of anti-drug antibodies and cause severe adverse effects [58]
Humanization	Only a few amino acids in the framework need to be exchanged, if necessary [59]	Exchanging or mutating Fc, CDR grafting on human framework region [60]
Generation	Immunization and PBMC isolation of camelids, sharks or transgenic mice, construction of an immune library and selection therefrom; or selection from a synthetic library [38,39,40,41,42,43]	Immunization and PBMC isolation of animals or humanized mice, or PBMC isolation from AIT-treated donors; fusion to myeloma (hybridoma) or sorting of B cells [32]
Production	In bacteria, yeast, plants, or eukaryotic cells [38]	In eukaryotic cells [32]
Costs	Depending on the expression system; production costs are generally lower in prokaryotic than in eukaryotic cells [38]	Depending on eukaryotic cells for production implies higher costs [32]

**Table 2:** Current status for nanobodies applied in allergy research and treatment.

Name/Reference	Target	Application	Current Status
Nb16 [51]	Ara h 3 (peanut)	Allergen detection	Developed
P43 [47]	Ara h 3 (peanut)	Allergen detection	Developed and validated
Nb82 [50]	β-Lactoglobulin (milk)	Allergen detection	Developed and validated
B91H/B40HA [48]	Lup an 1 (lupine)	Allergen detection	Developed and validated
Nb139H/Nb68HA [46]	Mac i 1 (macadamia)	Allergen detection	Developed and validated
VNAR14 [86,87]	Crustacean tropomyosin	Allergen detection	Developed and validated
AM1-1, AM1-3; AM2-A1, AM2-C2 [92]	Api m 1, Api m 2 (honey bee venom)	Allergy diagnostic	Developed, in vitro testing
G11, G24; G10 [93]	Phl p 4, Phl p 6 (timothy grass)	Allergy diagnostic	Developed, in vitro testing
Nb32, Nb32ILZ [44,45]	Bet v 1 (birch)	Allergy treatment	Developed, in vitro testing
Full HCAb [94,95]	IgE	Allergy treatment	Developed, in vitro testing, discontinued
ALX-0962 (sdab026) [79,96,97]	IgE	Allergy treatment	Developed, in vitro testing, discontinued
2IL43/2IL172/3ILT82 [80]	IL-13	Allergy treatment	Developed, in vitro testing
SAR443765 [98]	TSLP/IL-13	Allergy treatment	Clinical trial phase I completed
IL-5-HSA Nb [99]	IL-5/HSA	Allergy treatment	Preclinical phase

## Data Availability

Data are contained within the article.
